# Mode of Vaginal Delivery: A Modifiable Intrapartum Risk Factor for Obstetric Anal Sphincter Injury

**DOI:** 10.1155/2015/679470

**Published:** 2015-02-01

**Authors:** Marta Simó González, Oriol Porta Roda, Josep Perelló Capó, Ignasi Gich Saladich, Joaquim Calaf Alsina

**Affiliations:** ^1^Department of Gynecology and Obstetrics, Hospital de la Santa Creu i Sant Pau, Universidad Autónoma de Barcelona, C/Mas Casanovas 90, 08025 Barcelona, Spain; ^2^Clinical Epidemiology Unit, Hospital de la Santa Creu i Sant Pau, Universidad Autónoma de Barcelona, C/Mas Casanovas 90, 08025 Barcelona, Spain

## Abstract

The aim of this study was to analyze the comparative risks of this anal sphincter injury in relation to the type of intervention in vaginal delivery. We performed an observational, retrospective study of all vaginal deliveries attended at a tertiary university hospital between January 2006 and December 2009. We analyzed the incidence of obstetric anal sphincter injury for each mode of vaginal delivery: spontaneous delivery, vacuum, Thierry spatulas, and forceps. We determined the proportional incidence between methods taking spontaneous delivery as the reference. Ninety-seven of 4526 (2.14%) women included in the study presented obstetric anal sphincter injury. Instrumental deliveries showed a significantly higher risk of anal sphincter injury (2.7 to 4.9%) than spontaneous deliveries (1.1%). The highest incidence was for Thierry spatulas (OR 4.804), followed by forceps (OR 4.089) and vacuum extraction (OR 2.509). The type of intervention in a vaginal delivery is a modifiable intrapartum risk factor for obstetric anal sphincter injury. Tearing can occur in any type of delivery but proportions vary significantly. All healthcare professionals attending childbirth should be aware of the risk for each type of intervention and consider these together with the obstetric factors in each case.

## 1. Introduction

Obstetric anal sphincter injury encompasses third and fourth degree perineal tearing that occurs during delivery, according to Sultan's classification. This classification considers perineal injuries as a 3rd degree tear when there is any involvement of the anal sphincter and 4th degree tear when the anal epithelium is involved. This classification is incorporated in the RCOG guidelines and included in the Green Top Guidelines for the Management of Third and Fourth-Degree Perineal Tears Following Vaginal Delivery. Third degree tears are further classified into three subgroups according to the extent of damage to the external anal sphincter and internal anal sphincter [1, 2]. This classification is summarized in Table 1.

The incidence of obstetric anal sphincter injury varies between 0.5 and 5% of vaginal deliveries and it is the most common cause of anal incontinence in healthy women [3]. Obstetric anal sphincter injury is a serious complication of childbirth due to its notable maternal morbidity, its serious physical and emotional effects, and its impact on quality of life. Awareness of the factors most frequently associated with this injury is essential and can help obstetricians perform safer deliveries for both mother and child.

Many authors have studied the risk factors for obstetric anal sphincter injury and there is unanimity that its incidence is higher in occiput posterior and in instrumental deliveries with forceps [4]. Instruments used in the delivery room, however, can vary greatly between centers and countries. Most trials concerning instrumental deliveries considered forceps and vacuum in their analysis [5].

Spatulas are unarticulated instruments used mainly in French-speaking countries and a few other countries in Europe, Africa, and Latin American. The two most commonly used types of spatulas are Thierry and Teissier. Both types consist of two independent symmetric levers that are used to propel the fetal head forward, avoiding squeezing between the two branches. Teissier spatulas are shorter and less commonly used than the Thierry type [6]. Spatulas have classically been considered less aggressive than forceps on the basis of neonatal morbidity, and literature concerning their use and their maternal morbidity is scarce [7]. In daily clinical practice in our hospital, we use vacuum, forceps, and Thierry spatulas for instrumental deliveries. To our knowledge, no studies to date have compared all three interventions. The aim of this study was to analyze the comparative risk of obstetric anal sphincter injury in relation to the method used in instrumental deliveries.

## 2. Materials and Methods

We carried out an observational, retrospective study by analyzing data from the computerized database of all deliveries at Hospital de la Santa Creu i Sant Pau from January 1, 2006, until December 31, 2009. Our institution is a tertiary referral hospital in Barcelona, Spain. Personal identification details were omitted in all cases to ensure anonymity. The study was approved by the local Clinical Research Ethics Committee.

We included all vaginal deliveries of a singleton fetus in vertex presentation that occurred in our center during the study period. Exclusion criteria were birth by caesarean section, multiple births, and births with noncephalic presentations.

Our hospital is a training center for specialist physicians and midwives. Spontaneous deliveries can be attended by either physicians or midwives following a common protocol. The mother chooses the position in which she wants to give birth, but the perineum should always be visible to the attendant to permit “hands-on” perineal protection. Mediolateral episiotomy is only used when the attendant considers this necessary. Trainee physicians and midwives are closely overseen by a specialist in their field at all times. Instrumental vaginal deliveries are only performed by physicians. We perform and teach instrumental vaginal interventions using forceps, Thierry spatulas, and vacuum. The type of instrument is selected at the discretion of the attending physician, depending on the obstetric situation. When obstetric anal sphincter injury is diagnosed, primary repair is carried out in accordance with established guidelines.

The main outcome, obstetric anal sphincter injury, was classified according to the Sultan's classification [2]. Variables analyzed were type of delivery (spontaneous, vacuum, forceps, or Thierry spatulas), age, parity, anesthesia (epidural or local), type of labor onset (spontaneous or induction), duration of labor (hours from the beginning of the active phase of the first stage of labor until delivery), attendant (OBGYN trainee, OBGYN specialist, midwife, and midwife trainee), neonatal weight, and umbilical cord pH values.

The incidence of obstetric anal sphincter injury was analyzed according to the attendant and the type of vaginal delivery. Spontaneous vaginal birth was taken as the reference as it has the lowest incidence of this injury. We compared this with the other types of vaginal delivery (vacuum, forceps, and spatulas) rather than comparing the different types of instrumental delivery with each other.

### 2.1. Statistical Analysis

All variables were assessed in relation to the main outcome: obstetric anal sphincter injury. For categorical variables, the bivariate relationship was determined using contingency tables and inference with the corresponding chi-square or Fisher's exact test. For quantitative variables, the *t*-test for independent measurements was used. Multivariate analysis (logistic regression) was carried out. This analysis included all variables with a trend in the bivariate approach (*P* ≤ 0.10) or with clinical relevance. In all cases, the significance level used was the 5% (*α* = 0.05) with a bilateral approach. Analysis was performed using the SPSS Statistics V19.0.

## 3. Results

A total of 4526 vaginal births were recorded during the study period. Obstetric anal sphincter injury occurred in 97 cases, giving an incidence of 2.14% (CI 95% = 1.72–2.57).

Table 2 summarizes the different types of vaginal delivery. Our instrumentation rate was 31.31% for vaginal deliveries and 23.31% for all deliveries (including cesarean deliveries). According to the Sultan's classification, there were 93 3rd degree injuries (spontaneous 33, vacuum 3, forceps 22, and Thierry spatulas 35) and four 4th degree injuries (forceps 3 and Thierry spatulas 1).

Table 3 summarizes the results of the bivariate analysis. No significant differences were found between the obstetric anal sphincter injury and nonobstetric anal sphincter injury groups regarding maternal age, mode of onset of labor, duration of labor, or umbilical cord pH values at birth. We found that differences were statistically significant in the bivariate analysis for parity (*P* < 0.01), anesthesia (*P* = 0.026), attendant (*P* < 0.01), type of vaginal delivery (*P* < 0.01), and birth weight (*P* < 0.01). Neither parity nor anesthesia, however, was significant in the multivariate analysis, and both were excluded from the final model.  Table 4 shows the results obtained in the multivariate analysis.

The type of vaginal delivery (*P* < 0.01), the attendant (*P* < 0.01), and neonate birth weight (*P* < 0.01) showed statistically significant differences in the multivariate analysis. As neonatal weight is a nonmodifiable factor, we focused on the two factors in which we can intervene, the attendant and the type of vaginal delivery.

We analyzed the incidence of obstetric anal sphincter injury for each type of vaginal intervention. Women who had a spontaneous delivery had the lowest incidence of injury (1.1%), followed by vacuum extraction (2.7%) and forceps (4.5%). The use of Thierry's spatulas showed the highest incidence (4.9%). When comparing the differences in incidence of obstetric anal sphincter injury between spontaneous and instrumental delivery, we found significant results (*P* < 0.01). Vacuum extraction showed no significant differences (OR = 2.50). Forceps (OR = 4.08) and Thierry spatulas (OR = 4.80) showed statistically significant differences (*P* < 0.01). The risk of obstetric anal sphincter injury during delivery was fourfold higher using forceps and almost fivefold higher using Thierry spatulas than for spontaneous delivery.

The incidence of obstetric anal sphincter injury differed significantly in relation to the attendant (*P* < 0.01). OBGYN specialists showed a high incidence of injury (5.7%) compared to trainee physicians (2.3%), midwives (1.1%), and resident midwives (0.7%). It should be taken into account, however, that only physicians (both specialists and trainees) perform instrumental deliveries at our centre.

In terms of evaluation of the model, the Hosmer-Lemeshow test for goodness of fit showed no significant results (*P* = 0.548). The discrimination index (AUC-ROC) showed a value of 0.72, indicating good discrimination. We also calculated the ROC curve in the final model using 2 variables (neonatal weight and type of delivery) and 3 variables (adding attendant). The results showed similar values: 0.720 and 0.725.

## 4. Discussion

The main finding in this study was that the risk of obstetric anal sphincter injury differed in relation to the type of intervention in a vaginal delivery. Spatulas and forceps showed a significantly higher incidence of injury than spontaneous delivery and the incidence of injury was highest for Thierry spatulas.

Multivariate analysis identified the variables type of vaginal intervention, attendant, and neonatal weight as risk factors for obstetric anal sphincter injury in our group of patients. These results show special care should be taken when reviewing for tears so as to rule out obstetric anal sphincter injury in cases of high neonatal weight and in cases of instrumental delivery.

As neonatal weight is a nonmodifiable factor, we focus from here on the types of vaginal intervention during delivery and the attendant. In relation to vaginal intervention, we did not find many studies about the rate of instrumental delivery. In a multicentric study including data from 49 university hospitals in France in 2007, Mangin et al. published an interesting article about the rates of instrumental delivery. They concluded that the rate of operative delivery differed from one center to the other, ranging from 5.3 to 34.1% of all births [7]. In our setting of a university tertiary department where we perform and teach all types of instrumentation our rate of instrumentation (31.3% for vaginal deliveries and 23. 31% for all deliveries) could be considered within a common range.

Our results agree with earlier publications which found that several variables that were initially considered as risk factors for obstetric anal sphincter injury, such as primiparity, were later associated with instrumental extraction [8]. Episiotomy was not considered a variable due to the difficulty in credibly and objectively assessing this from the available data. Nevertheless, we do not consider our results were influenced by episiotomy because there is no consensus about the effect of this intervention with respect to tearing [9, 10].

The decision regarding the indication for instrumental delivery and the choice of instrument depended on the criteria of the attending physician. The fact that our analysis included all types of vaginal delivery gave us a wide view. Most studies to date have compared rates of obstetric anal sphincter injury for vacuum with rates for forceps. However, we compared the incidence of such injury in spontaneous vaginal delivery between three types of vaginal instrumentation: vacuum, spatulas, and forceps. We considered that this approach provided a new and more complete and objective picture of the real risk of each intervention.

Spontaneous delivery should be prioritized over other methods as an elective approach whenever feasible because of its lowest risk for this complication. The risk should be thoroughly evaluated when a delivery attendant considers a vaginal intervention is needed. The relative risk of obstetric anal sphincter injury for each type of intervention and its potential impact on the quality of life of the mother should be taken into account when selecting the instrument.

Our results support other studies showing that vacuum extraction has the lowest risk of obstetric anal sphincter injury among instrumental deliveries and should be considered, when obstetrically indicated, before other types of intervention. The higher risk of obstetric anal sphincter injury associated with the use of Thierry's spatula and forceps should be kept in mind when assessing their indication. Although spatulas may be considered less aggressive than forceps for neonatal morbidity, the risk for the mother must also be taken into account.

A final point worthy of consideration is the statistically significant difference found in relation to the attendant. OBGYN had the highest incidence of obstetric anal sphincter injury, but we did not consider this in the final statistical model. In our setting as a teaching hospital, OBGYN specialists intervene directly only in difficult deliveries. They supervise medical trainees and midwives but rarely intervene in spontaneous deliveries. Furthermore, midwives and midwife trainees never perform instrumental deliveries. For these reasons, we considered the results related to the variable “attendant” were biased. Nevertheless, we calculated the ROC curve using two models: with and without the attendant variable. The similar results support our argument that including attendant in the final model would not improve our results regarding the other two significant variables: neonatal weight and type of delivery.

In conclusion, all professionals attending a delivery of any type must be aware of the risks of obstetric anal sphincter injury. When an instrumental delivery is indicated, the most adequate approach should be carefully assessed, considering its potential impact. Such considerations could play a key role in reducing the incidence of obstetric anal sphincter injury and improving the maternal outcomes of childbirth.

## 5. Limitations and Strengths

Our study has several limitations. First, it was an observational, retrospective study of the register of our normal clinical practice, and occiput position or episiotomy was not systematically recorded in our database. Second, when intervention in a vaginal delivery was considered necessary, the decision concerning the type of intervention was left up to the specialist and, as in many operative and surgical techniques, this variable is difficult to standardize. The strengths of our paper are, however, the wide number of patients included and the comparison of the different modes of vaginal intervention. To our knowledge, these three interventions have not been compared together previously.

## Figures and Tables

**Table 1:** Sultan's classification of perineal trauma.

1st degree	Laceration of vaginal epithelium or perineal skin only	

2nd degree	Involvement of the perineal muscles but not the anal sphincter	

3rd degree	Disruption of the anal sphincter muscles	3a: <50% thickness of external sphincter torn
		3b: >50% thickness of external sphincter torn
		3c: internal sphincter torn

4th degree	Third degree tear with disruption of the anal epithelium as well	

**Table 2:** Types of vaginal delivery.

Type of delivery	Total (%)		ACC %

Spontaneous	3109 (68.69%)		68.69%

Vacuum	149 (3.29%)		31.31%
Forceps	553 (12.21%)	207 Kjelland (37.43%) 346 Naegele (62.57%)	

Thierry spatulas	715 (15.79%)	

Total	**4526 (100%)**		**100%**

**Table 3:** Summary of bivariate analysis results.

Variable	Categories	OASI	NO OASI	*P* value
Maternal age^**^	Years	31.33 (4.95)	31.02 (5.50)	*P* = 0.541

Parity^*^	Primipara	**70 (2.9%)**	**2324 (97%)**	**P** < 0.001
	Multipara	**27 (1.3%)**	**2104 (98.7)**	

Labor onset^*^	Spontaneous	75 (2%)	3622 (98%)	*P* = 0.287
	Induction	22 (2.7%)	807 (97.3%)	

Delivery duration	Hours	6.15 (3.64)	6.15 (5.04)	*P* = 0.995

Anesthesia^*^	Without	**8 (1.1%)**	**736 (98.9%)**	**P** = 0.026
	With	**89 (2.4%)**	**3693 (97.6%)**	

Type of delivery^*^	Spontaneous	**33 (1.1%)**	**3076 (98.9%)**	**P** < 0.001
	Vacuum extraction	**4 (2.7%)**	**145 (97.3)**	
	Forceps	**25 (4.5%)**	**528 (95.5%)**	
	Thierry's spatulas	**35 (4.9%)**	**680 (95.1%)**	

Assistant^*^	OBGYN	**18 (5.7%)**	**298 (94.3%)**	**P** < 0.001
	OBGYN trainee	**63 (2.3%)**	**2943 (97.7%)**	
	Midwife	**4 (1.1%)**	**349 (98.9%)**	
	Midwife trainee	**6 (0.7%)**	**822 (99.3%)**	

Neonatal weight^**^	Grams	**3438.35 (408.40)**	**3263.89 (508.93)**	**P** < 0.001

Umbilical cord pH^**^	Umbilical artery	7.22 (0.074)	7.23 (0.001)	*P* = 0.181

^*^Categorical variables: number and percentageof cases.

^**^Quantitative variables: mean and standard deviation.

**Table 4:** Summary of multivariate analysis results.

Variable			Coeff.	*P*	OR	CI 95% Lower OR	CI 95% Upper OR

Neonatal weight (grams)			0.001	0.002	1.001	1.000	1.001

Mode of delivery	Spontaneous delivery			*P* < 0.001	1^*^		
	Instrumental delivery	Vacuum	0.920	0.087	2.509	0.876	7.189
		Forceps	1.408	**<0.001**	**4.089**	2.406	6.949
		Thierry spatulas	1.569	**<0.001**	**4.804**	2.962	7.792

^*^Reference group.
